# In Vivo Optical Coherence Tomography Detection of Repetitive Plaque Erosion Leading to Healed Plaques and Lesion Progression

**DOI:** 10.1016/j.jaccas.2024.102693

**Published:** 2024-11-06

**Authors:** William Gibson, Elie Akl, Elvin Kedhi

**Affiliations:** Interventional Cardiology Department, Royal Victoria Hospital, McGill University Health Centre, Montréal, Québec, Canada

**Keywords:** Acute coronary syndrome, thrombosis, imaging, percutaneous coronary intervention, atherosclerosis

## Abstract

Plaque erosion is the second most common cause of acute coronary syndromes (ACS). Small studies using optical coherence tomography (OCT) have shown favorable outcomes in select patients with plaque erosion treated conservatively without stent implantation. Unlike plaque rupture, the role of plaque erosion in the formation of healed plaques and subsequent flow-limiting coronary stenoses is less certain. We present the case of a medically managed anterior ST-segment elevation myocardial infarction (STEMI) in a 53-year-old man, secondary to plaque erosion in the mid-left anterior descending (LAD) artery. Repeat OCT at 2 weeks demonstrated adequate resolution of intraluminal thrombus along with plaque layering with varying optical densities and negative invasive physiological testing. This case provides unique in vivo evidence of plaque erosion healing leading to the development of further plaque layering. We hypothesize that the multilayered plaque appearance after erosion is representative of repetitive episodes of plaque instability at the same coronary location, which may eventually lead to progression of plaque and reduction of lumen. Finally, there is mounting evidence that healed plaques represent an important predictor of future adverse events, raising important questions regarding the preconceived notion that plaque erosion has a benign course when treated conservatively.

## Graphical Abstract

**Figure:**
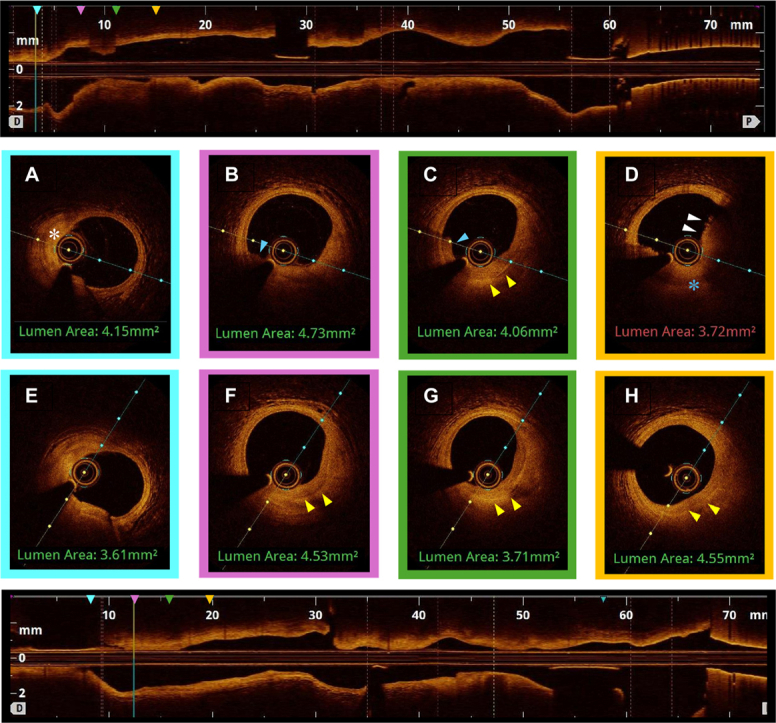

Plaque erosion is the second most important cause of ST-elevation myocardial infarction (STEMI) after plaque rupture, accounting for approximately one-third of all presentations.^1^^,^^2^ Although percutaneous coronary intervention (PCI) is the mainstay of treatment for plaque-rupture events, the definitive management of plaque erosion is less certain. Serial imaging studies using optical coherence tomography (OCT) suggest that a conservative approach with guideline-directed medical therapy and close angiographic follow-up, while avoiding angioplasty, may be safe and efficacious.^3^^,^^4^ Whether plaque erosion fully resolves or leads to a “healed plaque” is less well understood.

**Take-Home Messages**

• Our case emphasizes the value of using optical coherence tomography in the setting of acute coronary syndrome cases, wherein the underlying etiology is unclear.
• The optimal treatment approach to plaque erosion remains controversial, with some advocating for a conservative strategy.
• We identified plaque erosion causing acute coronary syndrome occurring at the site of a previously healed plaque and further confirm evidence of this healing process on follow-up imaging at 2 weeks.
• Overall, these findings highlight the need to further understand the significance of underlying plaque vulnerability in the context of plaque erosion and the implications for its management.

## Clinical Case

**Figure 1:**
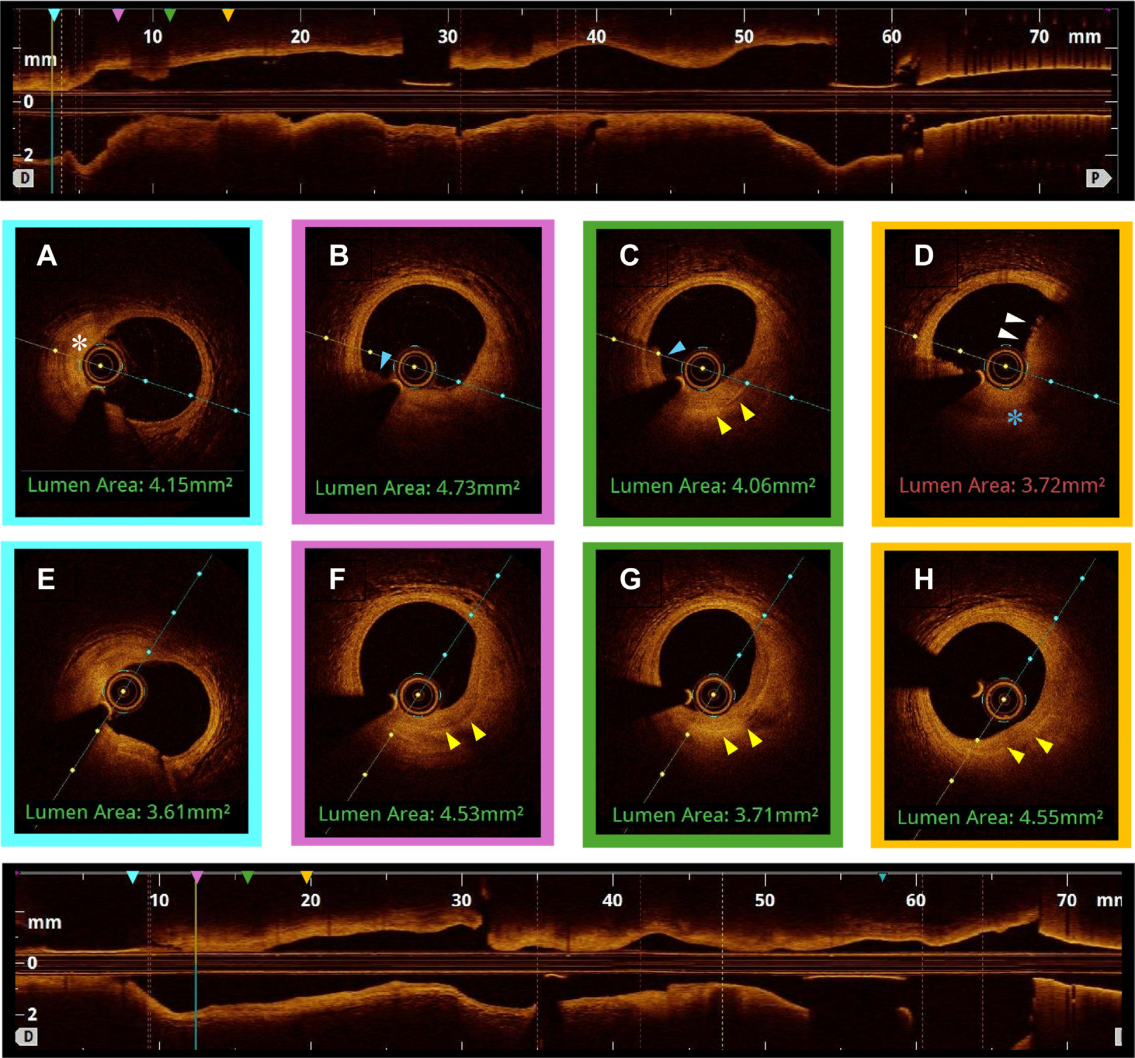
Index OCT and Follow-Up (A) Cross-sectional OCT image of distal reference (LAD) vessel showing fibroatheroma (white asterisk). (B, C) Layered plaque of varying optical densities (yellow arrowheads), suggestive of previous disruption or healing events, with small volume of overlying organized thrombus (blue arrowhead). (D) Organized thrombus (white arrowheads) in the absence of any obvious endothelial disruption (blue asterisk), indicating probable plaque. (E) Distal reference vessel. (F, G). Resolution of previous thrombus with apparent formation of new layer overlying previous healed plaque (yellow arrowheads). (H) Resolution of thrombus with layered plaque of various optical densities now apparent at this locus (yellow arrowheads), suggesting the occurrence of previous disruption and healing events. OCT = optical coherence tomography; LAD = left anterior descending.

**Figure 2:**
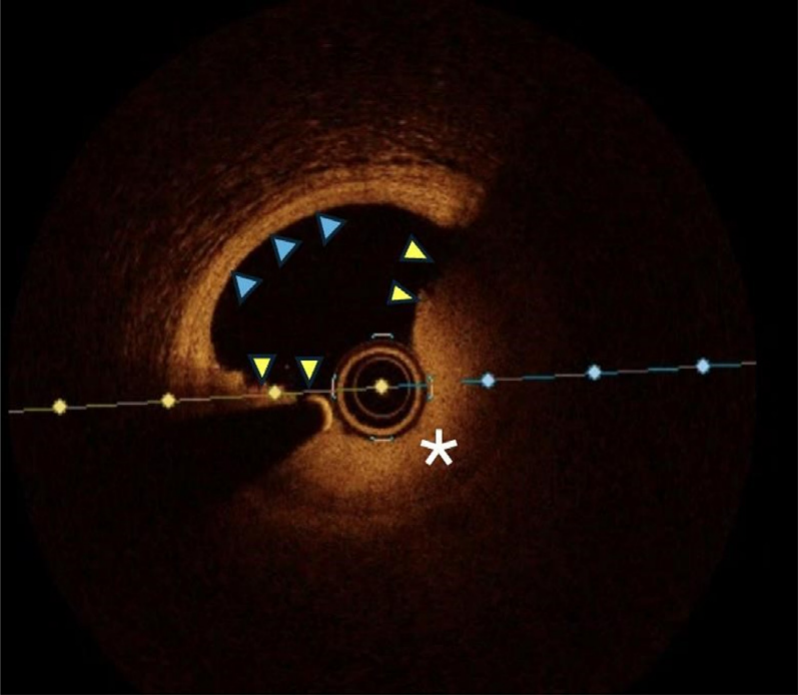
Index OCT Cross sectional OCT image of mid-LAD showing rather organized thrombus (yellow arrowheads) with high signal reflection and apparent integrity of the underlying fibrous cap (white asterisk) consistent with probably plaque erosion. Correspondingly healthy vessel wall is highlighted (blue arrowheads). Abbreviations as in Figure 1.

**Figure 3:**
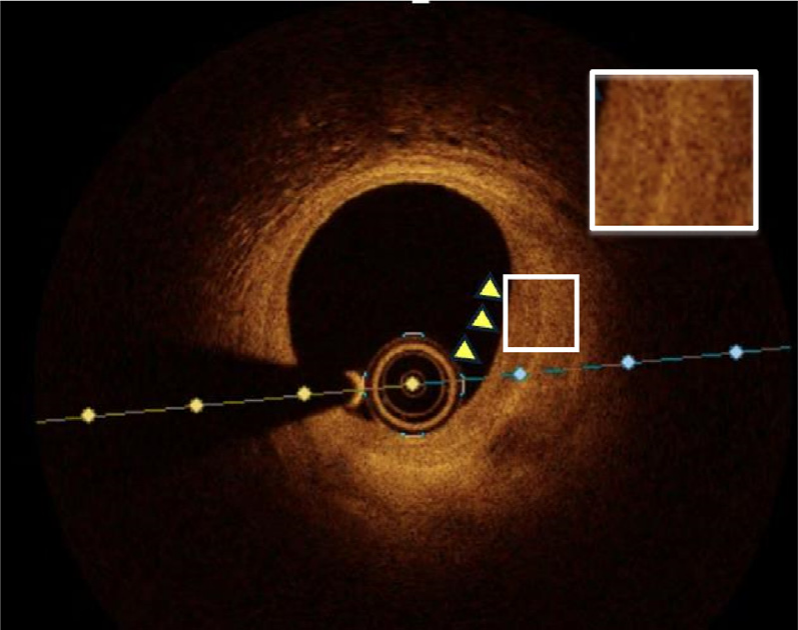
Follow-Up OCT at 2 Weeks Resolution of previous intraluminal thrombus and delineation of underlying plaque composed of multiple layers of varying optical densities (yellow arrowheads), suggestive of temporally distinct healed plaque events. Abbreviations as in Figure 1.

**Figure 4:**
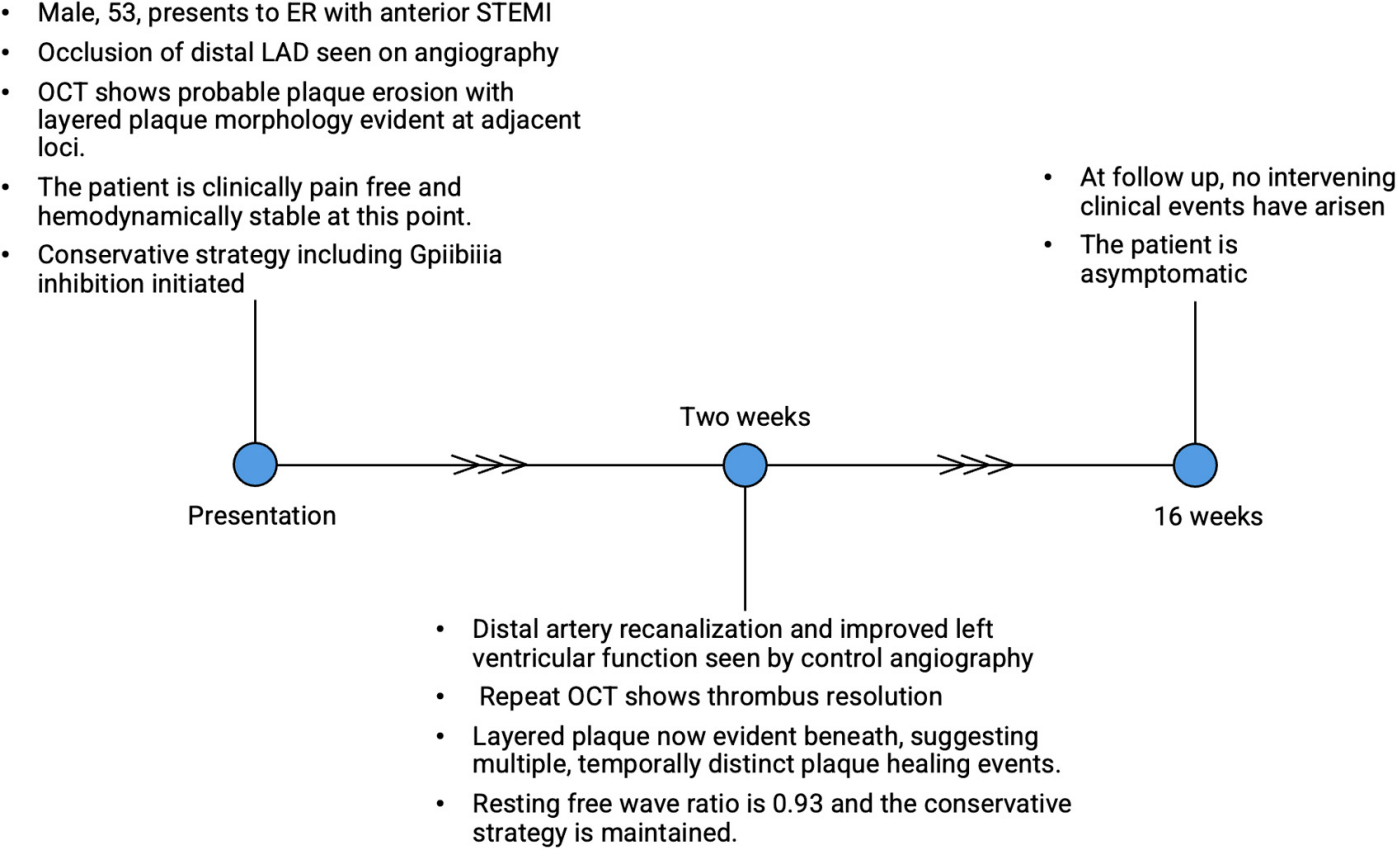
Timeline of Events ER = emergency room; STEMI = ST-segment elevation myocardial infarction; other abbreviations as in Figure 1.

We describe the case of a 53-year-old man who presented with an aborted anterior STEMI. Coronary angiography and left ventriculography revealed focal akinesis of the apex and an abrupt occlusion of the apical portion of the left anterior descending (LAD) artery. An intermediate lesion with haziness suggestive of possible intravascular thrombus was noted in the mid-segment. OCT confirmed the presence of relatively organized (red) thrombus without signs of cap disruption, suggesting probable plaque erosion (Figures 1D and 2) The percentage area of stenosis was 54%, and the minimum lumen area (MLA) was 2.94 mm^2^. Based on these findings and the absence of ongoing chest pain, the patient was treated conservatively with dual antiplatelet therapy, intravenous glycoprotein (GP) IIb/IIIa inhibitors, and unfractionated heparin, with planned angiographic control in 2 weeks. Repeat angiography revealed restoration of TIMI flow grade III^5^ flow to the apical LAD artery and partially improved apical wall motion. A resting full-cycle ratio (RFR) was performed, yielding a negative result (0.93). Repeat OCT imaging at this time showed no protruding thrombus but a layered plaque suggestive of plaque healing at the index culprit site (Figures 1 and 3), and a presumed new layer overlying a previously noted healed plaque were visible, whereas the lumen area at this point measured 3.71 mm^2^. Given the sufficient lumen patency, negative physiological assessment, and TIMI flow grade III, the conservative treatment plan was continued. Figure 4 depicts a timeline of the clinical course.

## Discussion

In recent years, the increasing use of intravascular imaging, particularly OCT, has greatly enhanced our understanding of plaque erosion as a distinct cause of acute coronary syndrome (ACS). Plaque erosion is characterized by predominantly white thrombus in the absence of identifiable intimal disruption on OCT. In contrast, plaque rupture is represented by thrombus in proximity to a visible disruption of the fibrous cap overlying mainly lipid-rich fibroatheromas. This distinction can be challenging on OCT in the presence of a significant thrombus burden, especially of the red type, which tends to obscure the visualization of the underlying intimal lining. Disrupted plaques with thrombus tend to heal by forming new tissue layers consisting of inflammatory cell infiltration, smooth muscle cells, and endothelial cells with surrounding extracellular matrix such as collagen and proteoglycans.^6^ Multiple layers with different optical densities and a clear demarcation from underlying components form what is called a multilayered plaque. Araki et al^7^ have shown that various modalities of plaque instability producing thrombus appear as “healed plaques” during follow-up. Histopathologic studies indicate that coronary atherosclerosis progresses in a phasic pattern secondary to recurrent, often clinically silent, thrombotic events caused by plaque disruption and subsequent healing at sites with vulnerable characteristics, including thin-cap fibroatheroma, large plaque volume, reduced minimal luminal area, and healed plaques.^8^ Healed plaques are increasingly recognized as a feature of plaque vulnerability and have been associated with future adverse clinical events.

Different OCT studies^9^^,^^10^ have shown that healed plaques are associated with a 2-fold risk of future adverse events. Del Val et al,^11^ in a subanalysis of the COMBINE trial, showed that healed plaques are the second most powerful predictor of future events after thin-cap fibroatheromas.

Compared with plaque rupture, the evidence for the interplay between plaque erosion, healed plaques, and luminal axial regression is less clear. Similarly, whether healed plaques following plaque erosion portend a similar risk of future clinical events is uncertain. A substudy of the EROSION (Effective Anti-Thrombotic Therapy Without Stenting: Intravascular Optical Coherence Tomography-Based Management in Plaque Erosion) trial, which identified newly formed healed plaques at the conservatively managed culprit site on OCT at 1 month, did not show an association between the presence of newly formed healed plaques and future clinical events.^12^ Although the invasive management of coronary atherosclerosis has primarily focused on treating obstructive lesions, the role of targeting nonobstructive lesions with vulnerable features is gaining traction. The recently published, randomized controlled PREVENT-MI trial showed a notable 3% absolute risk reduction in the primary endpoint of death, myocardial infarction (MI), ischemia-driven revascularization, and hospitalization for angina/ACS among patients treated with PCI for nonobstructive lesions (FFR >0.8) with evidence of plaque vulnerability compared with a conservatively managed control group.^13^ Although plaque erosion and rupture may be regarded as histopathologically distinct entities with potentially divergent therapeutic strategies, our case suggests that the underlying healing response may share similar characteristics with respect to progression of plaque,^7^^,^^8^ and each confer an increased risk of adverse future events. Furthermore, considering the potential myocardial damage occurring with each of these repetitive episodes, as clearly demonstrated in our case, it becomes questionable whether medical treatment is indeed better than mechanical treatment in these lesions, particularly in the setting of reduced minimum luminal area at presentation.

## Conclusions

Plaque erosion can occur repetitively within the same coronary loci, and their healing can lead to further progression of lumen. Whether medical rather than a mechanical treatment of these lesions can lead to improved clinical outcomes requires further exploration.

## Funding Support and Author Disclosures

Dr Kedhi has received institutional grants from Abbott and Medtronic; and proctorship funding from Abbott. All other authors have reported that they have no relationships relevant to the contents of this paper to disclose.
